# fMRI Evidence of Acupoints Specificity in Two Adjacent Acupoints

**DOI:** 10.1155/2013/932581

**Published:** 2013-05-23

**Authors:** Hua Liu, Jian-Yang Xu, Lin Li, Bao-Ci Shan, Bin-Bin Nie, Jing-quan Xue

**Affiliations:** ^1^Key Laboratory of Nuclear Analysis Techniques, Institute of High Energy Physics, Chinese Academy of Sciences, Beijing 100049, China; ^2^Beijing Engineering Research Center of Radiographic Techniques and Equipment, Beijing 100049, China; ^3^General Hospital of Chinese People's Armed Police Forces, Beijing 100039, China

## Abstract

*Objectives*. Acupoint specificity is the foundation of acupuncture treatment. The aim of this study is to investigate whether the acupoint specificity exists in two adjacent acupoints. *Design and Setting*. Two adjacent real acupoints, LR3 (Taichong) and ST44 (Neiting), and a nearby nonacupoint were selected. Thirty-three health volunteers were divided into three groups in random order, and each group only received acupuncture at one of the three points. While they received acupuncture, fMRI scan was performed. *Results*. The common cerebral activated areas responding to LR3 and ST44 included the contralateral primary somatosensory area (SI) and ipsilateral cerebellum. Acupuncture at LR3 specifically activated contralateral middle occipital gyrus, ipsilateral medial frontal gyrus, superior parietal lobe, middle temporal gyrus, rostral anterior cingulate cortex (rACC), lentiform nucleus, insula, and contralateral thalamus. Stimulation at ST44 selectively activated ipsilateral secondary somatosensory area (SII), contralateral middle frontal gyrus, inferior frontal gyrus, lingual gyrus, lentiform nucleus, and bilateral posterior cingulate cortex (PCC). *Conclusions*. Acupuncture at adjacent acupoints elicits distinct cerebral activation patterns, and those specific patterns might be involved in the mechanism of the specific therapeutic effects of different acupoints.

## 1. Introduction

Acupuncture, originated in ancient China, has been used as a treatment method in Asia for thousands of years. Nowadays, the therapeutic effect of acupuncture is gradually recognized in the western world. The National Institutes for Health of the United States have recommended acupuncture as an alternative and complementary treatment for many health conditions [1]. According to the traditional Chinese acupuncture theory as well as clinical practices, performance in specific acupoints can treat specific disorders. However, the exact physiological mechanism of acupuncture therapy is still unclear.

In the past decades, many studies of acupuncture on experimental animals have shown that acupuncture elicits therapeutic effect through modulating the neuroendocrine system [2]. Since 1990s, owing to the development of noninvasive brain imaging techniques such as functional MRI (fMRI) and positron emission tomography (PET), people have begun to address acupuncture investigation in human beings using functional imaging methods [3–6]. Siedentopf et al. reported that acupuncture at vision-related acupoints in the foot activated the visual association cortex with fMRI imaging [7]. Acupuncture at acupoints with strong analgesic effect, such as LI4 (Hegu), ST36 (Zusanli), and GB36 (Waiqiu), can modulate the hypothalamus and limbic system which are pain-related neuromatrix [8–12]. These results imply that the modulation effect of acupuncture might be related to the central nervous system. Moreover, acupuncture at specific acupoints could induce cerebral specific activation patterns. Our former work also shows there are specific cerebral patterns responding to different acupoints [13]. 

In the former studies on the acupoints specificity, the selected acupoints were generally far-between each other on the human body. In the current study, we chose two different and adjacently located acupoints to minimize the effect of general neural stimulation. If similar cerebral responses are derived from two real acupoints, the acupoints specificity needs to be further discussed. Otherwise, if significantly different activated areas are found, the theory of acupoint specificity will be supported. 

## 2. Design and Setting

### 2.1. Subjects

This study comprised 33 healthy right-handed volunteers (17 males and 16 females), aged 25.3 ± 2.8 (mean ± S.D.), without any history of psychiatric, neurological disorders, and substance abuse. All subjects had no acupuncture therapy experience. Each subject had provided informed consent with the adequate understanding of the procedure and purpose of this study. All subjects were free to withdraw from the experiment at any time. The protocol was approved by the local Ethics Committee. 

### 2.2. Stimuli

**Figure 1:**
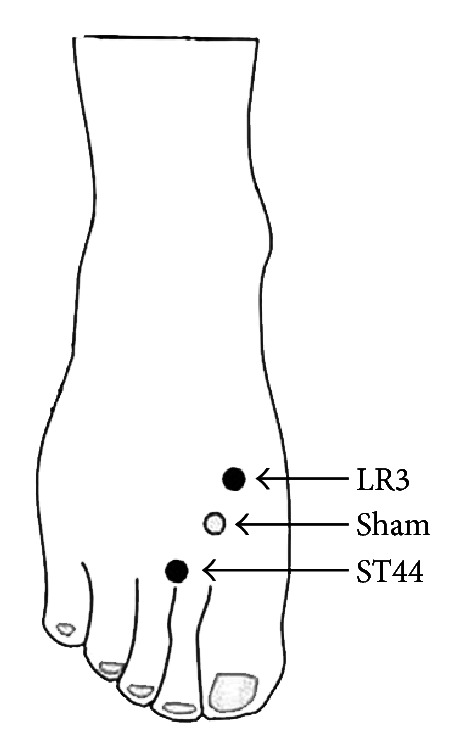
Anatomical location of the stimulation points: LR3, Taichong; ST44, Neiting; and their nearby nonacupoint.

Since manual acupoint stimulation is classical acupuncture, we adopted this acupuncture mode in this experiment. The silver needle is 0.30 mm in diameter and 25 mm in length. All acupuncture manipulations were performed by the same skilled acupuncturist. Two real acupoints and one nearby nonacupoint were selected in this experiment. The acupoint LR3 (Taichong) is located in the dorsum of the foot, in the depression anterior to the junction of the first and second metatarsals. The acupoint ST44 (Neiting) is located on the dorsum of the foot, proximal to the web margin between the second and third toes. Their nearby nonacupoint is located on the dorsum between the first and second metatarsals, approximately 10 mm anterolateral to LR3 and posteromedial to ST44 (Figure 1). The skilled acupuncturist identified that it was not located in any meridians.

All volunteers were divided into three groups in random order, and each group only received acupuncture at one of the three points. They were informed that they would receive acupuncture on the foot without being told the nature of the stimulation point. All the acupoints in this experiment were on right foot and anatomically innervated by the L5 spinal nerve.

### 2.3. Scanning Procedure

The experiments were performed on a 1.5 Tesla whole body scanner (Sonata, Siemens, Germany), with a standard head coil. The images covered whole brain and paralleled to the AC-PC line. Initially, the T_1_-weighted spin-echo images were obtained for anatomical reference. For the fMRI images, we employed a blood oxygenation level-dependent (BOLD) T_2_*-weighted gradient-echo EPI sequence with TR 3000 ms, TE 50 ms, flip angle 90°, field of view 220 mm × 220 mm, matrix 64 × 64, 6 mm slice thickness and 1.2 mm gap.

Given the fact that the therapeutic effect of acupuncture will last several minutes to several hours, which is called post effect, we adopted a single block design to avoid the influence of unknown duration of post effect [6, 13]. During scanning, subjects lay supinely on the scanner bed, keeping relaxed and calm. Their eyes were covered with blinders (Aearo Co., USA) and ears were plugged with earplugs (Aearo Co., USA). The lights in the scanning room were dimmed, and there were no sounds except scanner noise. When 62 baseline scans were finished, a sterile silver needle was inserted and twirled for 60 scans. Then the needle was withdrawn. While the scan continued, till total 402 scans were acquired. The needle was twirled manually clockwise and anticlockwise at about 1 Hz frequency with “even reinforcing and reducing” manipulation. The depth of needle insertion was approximately 15 mm for the real acupoint as well as the nonacupoint.

### 2.4. Data Analysis

The fMRI data were analyzed with statistical parametric mapping software (SPM2, Welcome Department of Imaging Neuroscience, London, United Kingdom, http://www.fil.ion.ucl.ac.uk/spm/). The first two images of each scan were discarded to avoid the nonequilibrium effects of magnetization, so every subject had 400 volumes. All volume images were automatically realigned to the first image of the time series to correct for head movement between scans. After realignment, the images were normalized and transformed into the Montreal Neurological Institute (MNI) space. Then spatial smoothing was done with a 9 mm × 9 mm × 9 mm Gaussian kernel. The smoothed data were processed with two levels. At the first level, each subject's data was, respectively, analyzed using fixed effect analysis based on the general linear model with a box-car reference waveform. The cerebral areas activated during acupuncture at the real acupoint and the nonacupoint relative to baseline were obtained. At the second level, in order to acquire the specific active areas induced by stimulating at the real acupoint compared to the nonacupoint, group analysis was performed using random effects analysis based on the two-sample *t*-test model with the results of first level (height threshold, *P* = 0.01 corrected, spatial extent threshold, 10 voxels). The coordinates in Talairach space were obtained by applying the Mattew Brett correction (mni2tal: http://imaging.mrc-cbu.cam.ac.uk/imaging/MniTalairach) to the SPM-MNI coordinates. 

## 3. Results

Deqi is a unique sensation of numbness, tingling, fullness, and dull ache that develops at the site of acupuncture and may spread some distance from the acupuncture point during needle manipulation. After scan, subjects were questioned as to the type and intensity of their psychophysical feeling to acupuncture. Based on their answers, all subjects who received stimulation at real acupoint experienced distinct sensation of Deqi. In all subjects who received stimulation at nonacupoint, only one subjects reported sensation of Deqi.

**Figure 2:**
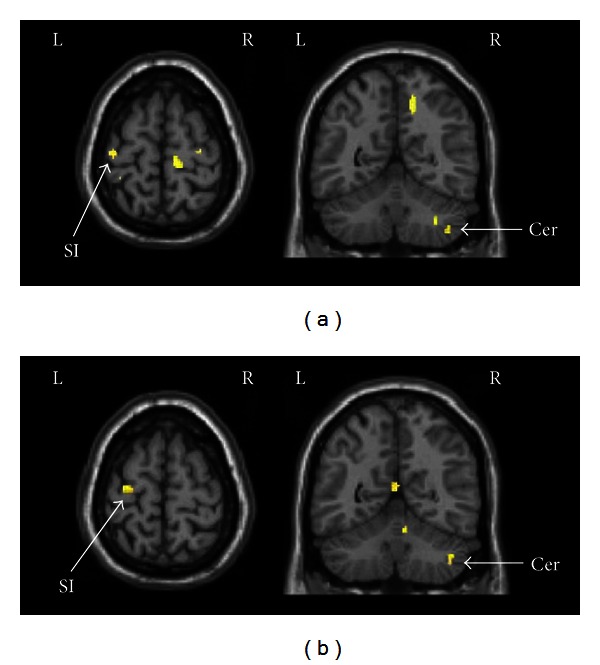
Common activated areas by acupuncture at LR3 or ST44. (a) Activation areas of LR3 versus nonacupoint. (b) Activation areas of ST44 versus nonacupoint. SI: primary somatosensory area; Cer: cerebellum.

**Figure 3:**
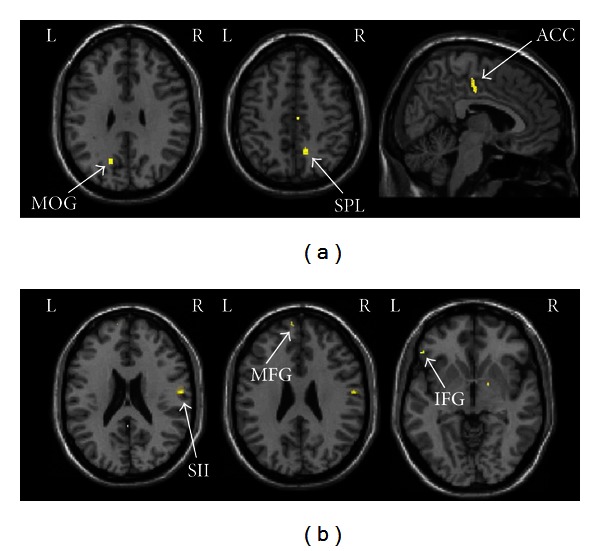
(a) Specific activated areas of LR3 contrasting to the nearby nonacupoint. (b) Specific activation areas of ST44 contrasting to the nearby nonacupoint. MOG: middle occipital gyrus; SPL: superior parietal lobe; ACC: anterior cingulate cortex; SII: secondary somatosensory area; MFG: middle frontal gyrus; IFG: inferior frontal gyrus.

Common areas activated by manual acupuncture on two real acupoints relative to nearby nonacupoint were illustrated in Figure 2, and specific areas activated by stimulation of LR3 or ST44 were showed in Figure 3. All results were summarized in Table 1. Acupuncture at LR3 significantly activated contralateral middle occipital gyrus (BA19), bilateral primary somatosensory area (SI), ipsilateral medial frontal gyrus (BA10), superior parietal lobe (BA7), middle temporal gyrus (BA21), rostral anterior cingulate cortex (rACC, BA24), lentiform nucleus, insula, cerebellum, and contralateral thalamus. Alternatively, acupuncture at ST44 selectively activated contralateral primary somatosensory area (SI), ipsilateral secondary somatosensory area (SII), lingual gyrus, lentiform nucleus, contralateral middle frontal gyrus (BA10), inferior frontal gyrus (BA47), bilateral posterior cingulate cortex (PCC, BA29), and cerebellum.

## 4. Discussion

In the present study, we applied manual acupuncture at two adjacent real acupoints and their nearby nonacupoint, which are all innervated by the same spinal segment, to further explore the acupoint specificity. The result showed that acupuncture at LR3 and ST44 elicited distinct response patterns, though they shared certain activation areas in common. 

The obvious overlapping activated areas of LR3 and ST44 were contralateral primary somatosensory area (SI) and ipsilateral cerebellum. The activations of these areas have also been reported by some previous studies on the acupuncture at other acupoints [12, 13, 15–17]. Nakagoshi et al. have acupunctured 6 acupoints, respectively, and summarized that SI might be partly responsible for acupuncture effect [18]. The same situation happened with cerebellum. Stimulation of acupoints might arouse the modulation effect of cerebellum beyond classical involvement of cerebellum in motor coordination [16]. 

Stimulation of LR3 selectively activated the middle occipital gyrus (BA19) which is considered as visual cortex. The study of Siedentopf et al. found that electroacupuncture at eye-related acupoints in the foot activated visual cortex [7]. Besides middle occipital gyrus (BA19), stimulation of LR3 also activated the medial frontal gyrus (BA10), superior parietal lobe (BA7), thalamus, and the limbic system. These areas were involved in visceral modulation [19]. It is worth to notice that LR3 were also very effective for visceral pain and body paralysis. The results confirmed the view that therapeutic effects of acupuncture may work through the central nervous system pathway.

Acupuncture at ST44 specifically activated superior frontal gyrus, inferior frontal gyrus and secondary somatosensory area (SII). Frontal areas are known to be related to pain [20], especially for abirritation of visceral pain. Activation of the SII cortex is thought to be related to the sensory-discriminative aspect of pain processing [21]. These areas have been reported in previous studies of pain treatment by acupuncture [13, 14]. In clinical practice, it is often used to cure toothache, sore throat, stomachache, swelling, and pain of dorsum of foot. This finding implied that acupuncture at ST44 may modulate activities of the frontal areas and SII cortex to inhibit pain.

Compared with previous experiment paradigm, our investigation chose two adjacent acupoints and their nearby nonacupoint to explore the acupoint specificity. Since we adopted two adjacent acupoints, the same nonacupoint could be available. The specific activated areas of two acupoints were acquired by contrasting the real acupoints with the same nonacupoint. Therefore, the effect of neural stimulation might be thoroughly eliminated from acupuncture stimulation. Our results might be more credible than previous studies.

## 5. Conclusions

In this study, results demonstrated that acupuncture at adjacent acupoints could elicit different fMRI activation patterns in the human brain. It is reasonable to suggest that acupuncture at different acupoints may modulate specific cerebral areas. Our results provide supplementary neuroimaging evidence for the existence of acupoint specificity. It is helpful to interpret the underlying mechanism of acupuncture.

## Figures and Tables

**Table 1:** Activated regions of brain induced by acupuncture at real acupoint versus nonacupoint.

Brain areas	Side	LR3 versus nonacupoint				ST44 versus nonacupoint			
		Talairach (mm)				Talairach (mm)			
		*X*	*Y*	*Z*	*Z*_max⁡_	*X*	*Y*	*Z*	*Z*_max⁡_
Primary somatosensory area	R	30	−14	60	2.54				
	L	−38	−14	60	2.45	−30	−13	60	3.09
Secondary somatosensory area	R					59	−9	21	3.03
Middle frontal gyrus	R	14	65	10	3.04				
	L					−8	63	23	2.71
Inferior frontal gyrus	L					−53	33	−5	2.98
superior parietal lobe	R	14	−50	47	3.16				
Middle temporal gyrus	R	61	−16	−14	2.78				
Middle occipital gyrus	R					28	−59	−7	2.68
	L	−18	−62	24	3.09				
Lingual gyrus	R					4	−79	−5	3.26
ACC	R	6	−15	41	2.81				
PCC	B					0	−48	15	3.26
Lentiform nucleus	R	28	−18	−1	3.42	18	0	−2	2.70
Thalamus	L	−12	−25	0	2.60				
Insula	R	32	−19	14	2.65				
Cerebellum	R	42	−56	−38	2.46	42	−52	−31	2.71
	L	−8	−64	−5	2.88	−24	−62	−29	3.55

Abbreviations: B: bilateral; R: right; L: left; ACC: anterior cingulate cortex; PCC: posterior cingulate cortex.
